# UNDERSTANDING OLDER AMERICANS’ DEBT: INSIGHTS FROM COGNITIVE AND IN-DEPTH INTERVIEWS

**DOI:** 10.1093/geroni/igae098.3147

**Published:** 2024-12-31

**Authors:** Hector Ortiz

**Affiliations:** Consumer Financial Protection Bureau, Washington, District of Columbia, United States

## Abstract

Debt is a common and important factor in older adults’ financial lives and balance sheets. According to the Survey of Consumer Finances, in 2022, 63 percent (31.3 million) of households headed by adults ages 60 and older held debt. In total, they held over $4.1 trillion in debt. Existing research and data suggest that there is also a strong relationship between debt and aging. This relationship is growing and changing. Despite the importance of debt in older adults’ financial lives, there are foundational gaps in qualitative data that limit researchers, policymakers, and educators’ ability to understand and monitor the level of debt held by older adults and the risk that debt poses to older adults. To address this gap, and provide insights into the growing and changing relationship between aging and debt, the Consumer Financial Protection Bureau (CFPB), an agency of the United States government, conducted cognitive and in-depth interviews with 54 participants, a majority (81 percent) of whom were age 62 or older, in June and July 2023. The findings revealed important gaps in the completeness of existing data sources that serve as a tool for monitoring trends and risks related to older adults’ debt. Moreover, the study findings also have important implications for areas where greater focus on consumer protection may be warranted.

